# Interim findings from first-dose mass COVID-19 vaccination roll-out and COVID-19 hospital admissions in Scotland: a national prospective cohort study

**DOI:** 10.1016/S0140-6736(21)00677-2

**Published:** 2021-05-01

**Authors:** Eleftheria Vasileiou, Colin R Simpson, Ting Shi, Steven Kerr, Utkarsh Agrawal, Ashley Akbari, Stuart Bedston, Jillian Beggs, Declan Bradley, Antony Chuter, Simon de Lusignan, Annemarie B Docherty, David Ford, FD Richard Hobbs, Mark Joy, Srinivasa Vittal Katikireddi, James Marple, Colin McCowan, Dylan McGagh, Jim McMenamin, Emily Moore, Josephine LK Murray, Jiafeng Pan, Lewis Ritchie, Syed Ahmar Shah, Sarah Stock, Fatemeh Torabi, Ruby SM Tsang, Rachael Wood, Mark Woolhouse, Chris Robertson, Aziz Sheikh

**Affiliations:** a Usher Institute, The University of Edinburgh, Edinburgh, UK; b Royal Infirmary of Edinburgh, NHS Lothian and Anaesthesia, Critical Care and Pain Medicine, The University of Edinburgh, Edinburgh, UK; c School of Health, Wellington Faculty of Health, Victoria University of Wellington, Wellington, New Zealand; d School of Medicine, University of St Andrews, St Andrews, UK; e Population Data Science, Swansea University Medical School, Swansea University, Swansea, UK; f Health Informatics, Health Informatics Group, College of Medicine, Swansea University, Swansea, UK; g The Health Data Research Hub for Respiratory Health, Edinburgh, UK; h Public Health Agency, Belfast, UK; i Centre for Public Health, Queen's University Belfast, Belfast, UK; j Nuffield Department of Primary Care Health Sciences, University of Oxford, Oxford, UK; k MRC/CSO Social & Public Health Sciences Unit, University of Glasgow, Glasgow, UK; l Public Health Scotland, Glasgow UK; m Clinical and Public Health Intelligence team, Public Health Scotland, Edinburgh, UK; n Department of Mathematics and Statistics, University of Strathclyde, Glasgow, UK; o Academic Primary Care, University of Aberdeen School of Medicine and Dentistry, Aberdeen, UK

## Abstract

**Background:**

The BNT162b2 mRNA (Pfizer–BioNTech) and ChAdOx1 nCoV-19 (Oxford–AstraZeneca) COVID-19 vaccines have shown high efficacy against disease in phase 3 clinical trials and are now being used in national vaccination programmes in the UK and several other countries. Studying the real-world effects of these vaccines is an urgent requirement. The aim of our study was to investigate the association between the mass roll-out of the first doses of these COVID-19 vaccines and hospital admissions for COVID-19.

**Methods:**

We did a prospective cohort study using the Early Pandemic Evaluation and Enhanced Surveillance of COVID-19—EAVE II—database comprising linked vaccination, primary care, real-time reverse transcription-PCR testing, and hospital admission patient records for 5·4 million people in Scotland (about 99% of the population) registered at 940 general practices. Individuals who had previously tested positive were excluded from the analysis. A time-dependent Cox model and Poisson regression models with inverse propensity weights were fitted to estimate effectiveness against COVID-19 hospital admission (defined as 1–adjusted rate ratio) following the first dose of vaccine.

**Findings:**

Between Dec 8, 2020, and Feb 22, 2021, a total of 1 331 993 people were vaccinated over the study period. The mean age of those vaccinated was 65·0 years (SD 16·2). The first dose of the BNT162b2 mRNA vaccine was associated with a vaccine effect of 91% (95% CI 85–94) for reduced COVID-19 hospital admission at 28–34 days post-vaccination. Vaccine effect at the same time interval for the ChAdOx1 vaccine was 88% (95% CI 75–94). Results of combined vaccine effects against hospital admission due to COVID-19 were similar when restricting the analysis to those aged 80 years and older (83%, 95% CI 72–89 at 28–34 days post-vaccination).

**Interpretation:**

Mass roll-out of the first doses of the BNT162b2 mRNA and ChAdOx1 vaccines was associated with substantial reductions in the risk of hospital admission due to COVID-19 in Scotland. There remains the possibility that some of the observed effects might have been due to residual confounding.

**Funding:**

UK Research and Innovation (Medical Research Council), Research and Innovation Industrial Strategy Challenge Fund, Health Data Research UK.

## Introduction

In December, 2019, there was an outbreak of the novel SARS-CoV-2 in Wuhan, China, which was later declared as the COVID-19 pandemic by WHO. Since the beginning of the pandemic and as of April 20, 2021, more than 141 million cases and 3 millions deaths have been reported in more than 223 countries and territories worldwide. The UK has among the highest morbidity and mortality rates worldwide. Scotland has reported more than 24 000 hospital admissions and 7600 deaths due to COVID-19.^1^

Unprecedented investments have been made in vaccine technology, evaluation, and production in response to the pandemic. Authorisation of the first COVID-19 vaccines occurred soon after publication of the initial phase 3 safety and efficacy studies,^2^ and the UK was one of the first countries to license these vaccines for use.^1^ As of April 13, 2021, first-dose vaccine coverage of about 50% has been reported in Scotland with more than 2·6 million vaccines administered across the Scottish population, and delivery targeted at specified priority groups of those most at risk of harm (including those aged 50 years and older and health-care workers; appendix p 1).^1^, ^3^

Clinical trials of all three vaccines authorised for use in the UK (ie, Pfizer–BioNTech, Oxford–AstraZeneca, and Moderna) have reported high vaccine efficacy. For the Pfizer–BioNTech vaccine (BNT162b2 mRNA COVID-19 vaccine), 95% efficacy was reported against laboratory-confirmed COVID-19.^4^ The Oxford–AstraZeneca vaccine was found to have 70% efficacy against COVID-19 illness among seronegative participants.^5^ The Moderna vaccine (mRNA-1273) was reported to have 94% efficacy against confirmed COVID-19 but, given that the first dose of mRNA-1273 was given in Scotland on April 7, 2021, it is therefore not included in this analysis.^6^

**Research in context**

**Evidence before this study**

We searched PubMed, medRxiv, and SSRN for observational studies, with no language restrictions, using the term “COVID-19 vaccine effect”. We searched for studies published between Dec 1, 2020, and March 2, 2021. We found one study from Israel, which included 596 618 individuals who received the BNT162b2 mRNA (Pfizer–BioNTech) vaccine and an equal number of controls. The study showed that 14–20 days after the first vaccination dose there was a vaccine effect of 74% (95% CI 56–86) for hospital admissions due to COVID-19. Public Health England has reported that the first dose vaccine effect against hospital admissions due to COVID-19 for the BNT162b2 mRNA and ChAdOx1 nCoV-19 (Oxford–AstraZeneca) vaccines is about 80%.

**Added value of this study**

Current Scottish (UK) policy for use of vaccines against COVID-19 involves an offer of a first dose followed by a second dose 12 weeks later. To our knowledge, this is the first study of COVID-19 vaccine effect against hospital admissions for an entire nation after a single dose of vaccine. We found that a single dose of the BNT162b2 mRNA COVID-19 vaccine was associated with a vaccine effect of 91% (95% CI 85–94) for hospital admissions due to COVID-19 28–34 days after vaccination. A single dose of ChAdOx1 vaccine was associated with a vaccine effect of 88% (95% CI 75–94) at 28–34 days post-vaccination. Comparable vaccine effects were seen in those aged 80 years and older against hospital admissions due to COVID-19 with a high combined vaccine effect of 83% (95% CI 72–89) at 28–34 days after vaccination. For the same age group (≥80 years old) and at the same post-vaccination period (28–34 days), a vaccine effect of 88% (95% CI 76–94) was found for the BNT162b2 mRNA vaccine and 81% (60–91) for the ChAdOx1 vaccine.

**Implications of all the available evidence**

We provide national evidence that the mass roll-out of first doses of the COVID-19 vaccines currently being used in the UK vaccination programme was associated with substantial reductions in risk of COVID-19 hospital admissions in the populations at highest risk for severe COVID-19 outcomes. However, we note that some of the observed effects might have been due to residual confounding.

Large post-licensure epidemiological studies are needed to complement the findings of pre-licensure trials to estimate the effectiveness of these vaccines at the population level in real-world settings.^7^ The delayed second dose COVID-19 vaccination policy of the UK does not comply with the manufacturer guidance on timing between the first and second dose (ie, 21 days for Pfizer–BioNTech and 28 days for Oxford–AstraZeneca vaccines). Reflecting the need to gather evidence on this policy, we aimed to investigate the association between the first doses of the Pfizer–BioNTech and Oxford–AstraZeneca vaccines and hospital admissions in those with confirmed COVID-19 among high-risk adults in Scotland.

## Methods

### Study design and participants

We did an open, real-time prospective observational cohort study with national-level coverage in Scotland using a unique dataset consisting of linked vaccination, primary care, laboratory testing, hospital admission, and mortality data (appendix p 3). Data were available for 5·4 million people in Scotland.^8^ Individuals who had previously tested positive with real-time reverse transcription-PCR (rtPCR) for SARS-CoV-2 infection before Dec 8, 2020, were excluded from this analysis. Ethical approval was obtained from the National Research Ethics Service Committee, Southeast Scotland 02 (reference number, 12/SS/0201), and Public Benefit and Privacy Panel for Health and Social Care (reference number, 1920–0279). We produced a protocol before undertaking the analysis. We followed the Reporting of studies Conducted using Observational Routinely-collected Data^9^ checklist to guide transparent reporting of this cohort study (appendix pp 16–23). Our analysis code has been made publicly available.

### Procedures

By use of the Early Pandemic Evaluation and Enhanced Surveillance of COVID-19—EAVE II—database, primary care data derived from 940 general practices across Scotland were linked to the laboratory data from the Electronic Communication of Surveillance in Scotland (ECOSS),^8^ the hospital admission data available from the Scottish Morbidity Record 01 database, and Rapid Preliminary Inpatient Data.^10^ Vaccination data were available from general practices and the Turas Vaccination Management Tool (TVMT),^11^ which is a web-based tool to capture vaccinations in the community and create real-time vaccination records. Laboratory data from ECOSS included all rtPCR test results from both National Health Service laboratories (Pillar 1) and Lighthouse Government laboratories (Pillar 2).^12^ Data were deterministically linked using the Community Health Index number, which is a unique identifier used for all health-care contact across Scotland.^8^

We studied the first doses of the BNT162b2 mRNA COVID-19 (also known as the Pfizer–BioNTech) vaccine^4^ and ChAdOx1 (AZD1222; also known as the Oxford–AstraZeneca) vaccine.^5^ An individual was defined as exposed if they received a single dose of vaccine between Dec 8, 2020, and Feb 22, 2021, with maximum follow-up time censored at Feb 22, 2021 (the latest event date). Vaccinated groups were stratified by time intervals including 0–6, 7–13, 14–20, 21–27, 28–34, 35–41, and 42 or more days post-vaccination, and by the type of vaccine received. Vaccination information was extracted from the general practitioner records and the TVMT system and included individuals vaccinated in general practices, community vaccination hubs, and other settings such as care homes.

The primary outcome was vaccine effect, assessed as hospital admissions with COVID-19 as the main cause of admission, or hospital admission within 28 days of a positive rtPCR test for SARS-CoV-2 infection from Dec 8, 2020, to Feb 22, 2021. International Classification of Diseases-10 codes used for COVID-19 illness are presented in the appendix (p 5). A post-hoc sensitivity analysis was also carried out to assess any vaccine programme effects or residual confounding effects during the early post-vaccination period. An additional post-hoc exploratory ecological analysis was done to investigate the effect of lockdown measures on hospital admissions between September, 2020, and February, 2021.

### Statistical analysis

The primary analyses included vaccine effect estimates for vaccination status overall and for each vaccine type. The secondary analysis included vaccine effect estimates for vaccine status overall and for each vaccine type stratified by age groups (ages 18–64, 65–79, and ≥80 years). These were grouped to the completed year (ie, 64·5 years would be categorised as 18–64 years and 79·4 as 65–79 years).

Baseline characteristics in the vaccinated and unvaccinated groups were described using proportions. We assessed the effect of one dose of either vaccine against hospital admissions due to laboratory-confirmed SARS-CoV-2 infection, or clinical diagnosis of COVID-19 on admission. Poisson regression adjusting for an offset representing the time at risk and time-dependent Cox models (considering the time at risk) were used to derive the rate ratios, hazard ratios, and 95% CIs for the association of vaccination with COVID-19 hospital admissions.

Cox models included spline terms for age and number of rtPCR tests before vaccination (a marker for health-care workers, social-care workers, and care home residents who had repeated tests). Additional adjustments were made for sex, socioeconomic status, and underlying medical conditions at risk of COVID-19 illness with vaccination groups representing a time-dependent covariate. Calendar time intervals by week were included as stratification variables because the background epidemic was changing rapidly over the observation period. Poisson regression was used for the full adjustment and propensity weighting. This regression model used age groups in 5-year intervals as well as sex, deprivation, and number of previous tests. Additionally, the following comorbidity groups, all of which are associated with an increased risk of hospital admission, were included: type 1 and type 2 diabetes, high and low blood pressure, chronic obstructive pulmonary disease, chronic kidney disease, dementia, stroke, learning disorders, fractures, neurological conditions, chronic cardiac failure, asthma, epilepsy, blood cancer, liver cirrhosis, venous thromboembolism, peripheral vascular disease, atrial fibrillation, pulmonary hypertension, Parkinson's disease, rare pulmonary disorders, rheumatoid arthritis, and systemic lupus erythematosus.

Both the Cox models and Poisson regression used sampling weights, which were used to correct for the size of the registered general practice population being greater than the population in Scotland (some due to recently deceased patients still being recorded in the patient records and individuals who had recently moved). These weights were derived by matching the age and sex numbers in the general practice data to the Scottish population data. This adjustment ensured that the denominators in the tables matched the Scottish population.

The models were fit to a dataset with all events and a random sample, without replacement, of 100 individuals per event with sample weights calculated to represent the sampling fraction and thus ensure the correct calculation of the person-years at risk for the whole population. A combined weight was used in the statistical modelling. Finally, a propensity model for vaccination was developed using a logistic regression model including terms for age group by sex interaction, socioeconomic status, number of previous rtPCR tests, and number of clinical risk groups. The parameter estimates of this model were obtained from a 25% sample of the cohort and the propensity score calculated for the whole cohort. Inverse propensity score weights were used as a final adjustment in the regression models.^13^

33 834 individuals received a second dose of the vaccine. Individuals who received two doses remained in the analysis for as long as they had one dose and were then censored at the date of the second dose. All statistical tests were two-tailed with a 5% significance level of p less than 0·05.

At the baseline of our cohort (Dec 8, 2020), several population characteristics that could potentially confound the association between COVID-19 vaccination and the outcomes of interest were determined. These included age, sex, socioeconomic status measured by quintiles of the Scottish Index of Multiple Deprivation (1 refers to most deprived and 5 refers to least deprived),^8^ residential settlement measured by the urban or rural 6-fold classification (1 refers to large urban areas and 6 refers to small remote rural areas),^8^ and the number and types of comorbidities commonly associated with COVID-19 illness.^8^ To adjust for the residual confounding in which vaccines were not offered to or were declined by the most frail, we included a functional variable, namely dementia, in our covariate adjustment.^14^

We did several post-hoc sensitivity analyses to assess possible vaccine programme or residual confounding effects during the early period after vaccination (ie, <14 days when immunity should still be mounting).^4^ In particular, we carried out a falsification of exposure sensitivity analysis that involved re-running the analysis using a fictional date of vaccination 2 months before actual vaccination to determine the extent to which vaccine programme effects contributed to residual confounding. We have provided the overall predicted curves from the Cox proportional hazards model adjusting for covariates, with a stratification by calendar period (appendix pp 6–7). An over-dispersed Poisson regression model was also carried out to assess the effect of the national lockdown on Dec 26, 2020, and the additional restrictions imposed on Jan 5, 2021, on hospital admissions due to COVID-19 in adults (age 18–64, 65–79, and 80 or older).

The analyses were carried out by one statistician (CR) and independently checked by a second statistician (EM). All statistical analyses were done with statistical software R (version 3.6.1).

There was missing data for deprivation, urban-rural status (patient postcode was not available), smoking status, and blood pressure (value had not been recorded). Missing data were handled by creating a separate group for these individuals.

### Role of the funding source

The funder of the study had no role in study design, data collection, data analysis, data interpretation, or the writing of the report.

## Results

**Figure 1:**
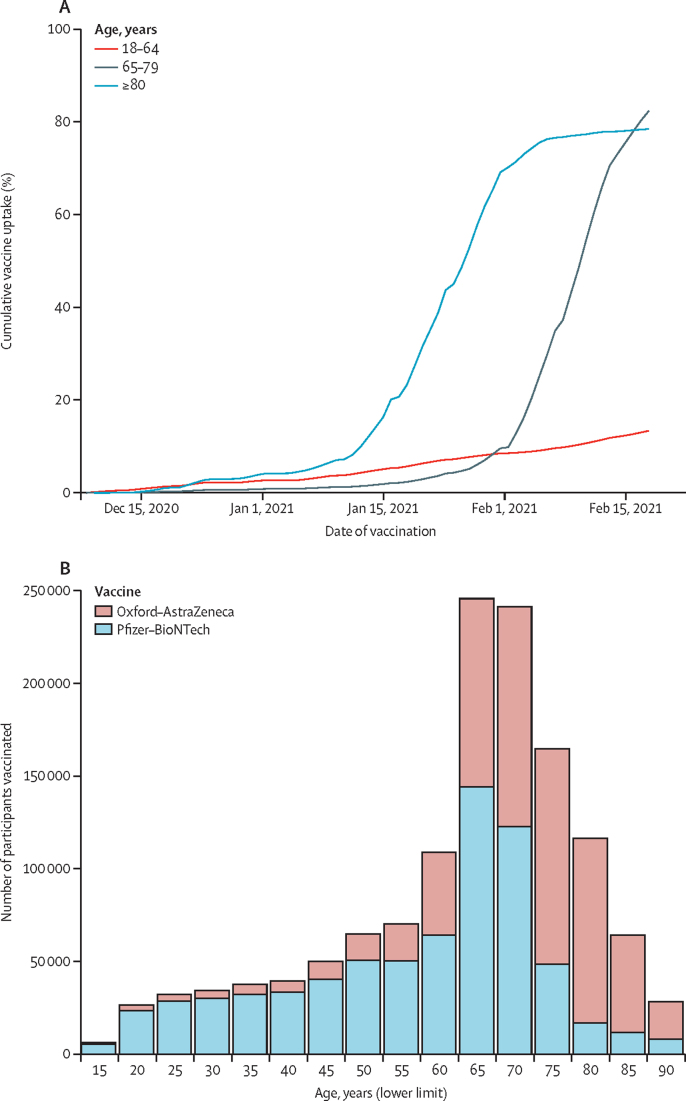
COVID-19 vaccine uptake (A) Uptake over time by age group. (B) Uptake by age and vaccine type.

Between Dec 8, 2020, and Feb 15, 2021, 1 331 993 (30%) of 4 409 588 adults aged 18 years or older were vaccinated in our study. Rapid uptake of the BNT162b2 mRNA and ChAdOx1 vaccines was observed over the study period (figure 1; table 1), with 65–79 years the age group with the highest uptake (85·9%; 651 942 of 758 946). For the BNT162b2 mRNA vaccine, the highest uptake was found in patients younger than 65 years, whereas for the ChAdOx1 vaccine, highest vaccine uptake was found in patients aged 65–79 years (table 1). Vaccine uptake for both vaccines combined was higher in women (35·1%, 799 765 of 2 280 907 *vs* 25·0%, 532 228 of 2 128 681 men). A low socioeconomic status was associated with lower vaccination rates than was a high socioeconomic status (level 1, 26·1% *vs* levels 3–5, 31·2–32·0%). Those living in large urban areas had an uptake of 25%, which was lower than all other residential categories. Vaccine coverage increased with the number of comorbidities, from 21·2% with none to 80·0% with five or more. Ex-smokers had a coverage of 48·7%, which was higher than the similar uptake recorded for both smokers and non-smokers. Vaccine coverage also increased with increasing blood pressure. These differences in the clinical factors were probably associated with age. Baseline characteristics of participants older than 80 years by vaccine status and timing are presented in the appendix (pp 10–12).

**Table 1:** Baseline characteristics by vaccine status

	**Vaccinated**			**Unvaccinated (n=3 077 595)**	**Uptake (% of total for both vaccines)**
	Both vaccines (n=1 331 993)	BNT162b2 (Pfizer–BioNTech) (n=711 839)	ChAdOx1 (Oxford–AstraZeneca) (n=620 154)		
**Sex**					
Female	799 765 (60·0%)	456 667 (64·2%)	343 098 (55·3%)	1 481 142 (48·1%)	799 765/2 280 907 (35·1%)
Male	532 228 (40·0%)	255 172 (35·8%)	277 056 (44·7%)	1 596 453 (51·9%)	532 228/2 128 681 (25·0%)
**Age group, years**					
18–64	470 960 (35·4%)	359 434 (50·5%)	111 526 (18·0%)	2 913 484 (94·7%)	470 960/3 384 444 (13·9%)
65–79	651 924 (48·9%)	315 620 (44·3%)	336 304 (54·2%)	107 022 (3·5%)	651 924/758 946 (85·9%)
≥80	209 109 (15·7%)	36 785 (5·2%)	172 324 (27·8%)	57 089 (1·9%)	209 109/266 198 (78·6%)
Socioeconomic status*					
1	225 951 (17·0%)	126 858 (17·8%)	99 093 (16·0%)	640 087 (20·8%)	225 951/866 038 (26·1%)
2	259 785 (19·5%)	140 337 (19·7%)	119 448 (19·3%)	606 554 (19·7%)	259 785/866 339 (30·0%)
3	279 616 (21·0%)	142 783 (20·1%)	136 833 (22·1%)	593 487 (19·3%)	279 616/873 103 (32·0%)
4	283 288 (21·3%)	151 265 (21·2%)	132 023 (21·3%)	592 476 (19·3%)	283 288/875 764 (32·0%)
5	276 680 (20·8%)	146 461 (20·6%)	130 219 (21·0%)	609 927 (19·8%)	276 680/886 607 (31·2%)
Unknown	6673 (0·5%)	4135 (0·6%)	2538 (0·4%)	35 064 (1·1%)	6673/41 737 (16·0%)
**Residential settlement**†					
1	398 251 (29·9%)	217 396 (30·5%)	180 855 (29·2%)	1 192 363 (38·7%)	398 251/1 590 644 (25·0%)
2	499 274 (37·5%)	279 933 (39·3%)	219 341 (35·4%)	1 052 938 (34·2%)	499 274/1 552 212 (32·2%)
3	133 842 (10·0%)	68 411 (9·6%)	65 431 (10·6%)	269 309 (8·8%)	133 842/403 151 (33·2%)
4	79 993 (6·0%)	36 623 (5·1%)	43 370 (7·0%)	131 373 (4·3%)	79 993/211 366 (37·8%)
5	129 389 (9·7%)	62 956 (8·8%)	66 433 (10·7%)	263 175 (8·6%)	129 389/392 564 (33·0%)
6	84 571 (6·3%)	42 385 (6·0%)	42 186 (6·8%)	133 373 (4·3%)	84 571/217 944 (38·8%)
Unknown	6673 (0·5%)	4135 (0·6%)	2538 (0·4%)	35 064 (1·1%)	6673/41 737 (16·0%)
**Number of comorbidities**					
0	561 329 (42·1%)	358 088 (50·3%)	203 241 (32·8%)	2 086 239 (67·8%)	561 329/2 647 568 (21·2%)
1	379 133 (28·5%)	201 507 (28·3%)	177 626 (28·6%)	723 055 (23·5%)	379 133/1 102 188 (34·4%)
2	204 988 (15·4%)	86 722 (12·2%)	118 266 (19·1%)	192 945 (6·3%)	204 988/397 933 (51·5%)
3	102 835 (7·7%)	37 374 (5·3%)	65 461 (10·6%)	51 005 (1·7%)	102 835/153 840 (66·8%)
4	49 289 (3·7%)	16 700 (2·3%)	32 589 (5·3%)	15 763 (0·5%)	49 289/65 052 (75·8%)
≥5	34 419 (2·6%)	11 448 (1·6%)	22 971 (3·7%)	8589 (0·3%)	34 419/43 008 (80·0%)
**Type of comorbidity**					
Asthma	175 171 (13·2%)	91 175 (12·8%)	83 996 (13·5%)	384 091 (12·5%)	175 171/559 262 (31·3%)
Chronic kidney condition‡ (stage 3)	133 393 (10·0%)	41 172 (5·8%)	92 221 (14·9%)	28 140 (0·9%)	133 393/161 533 (82·6%)
Liver cirrhosis	11 821 (0·9%)	5075 (0·7%)	6746 (1·1%)	11 518 (0·4%)	11 821/23 339 (50·6%)
Chronic neurological condition	8395 (0·6%)	3413 (0·5%)	4982 (0·8%)	9718 (0·3%)	8395/18 113 (46·3%)
Heart failure	35 998 (2·7%)	11 016 (1·5%)	24 982 (4·0%)	12 103 (0·4%)	35 998/48 101 (74·8%)
Diabetes (type 1)	6840 (0·5%)	3651 (0·5%)	3189 (0·5%)	14 582 (0·5%)	6840/21 422 (31·9%)
Diabetes (type 2)	156 720 (11·8%)	64 426 (9·1%)	92 294 (14·9%)	101 821 (3·3%)	156 720/258 541 (60·6%)
Dementia	32 317 (2·4%)	16 993 (2·4%)	15 324 (2·5%)	5493 (0·2%)	32 317/37 810 (85·5%)
Coronary heart disease	146 465 (11·0%)	52 895 (7·4%)	93 570 (15·1%)	55 643 (1·8%)	146 465/202 108 (72·5%)
**Smoking status**					
Ex-smoker	277 240 (20·8%)	123 234 (17·3%)	154 006 (24·8%)	291 792 (9·5%)	277 240/569 032 (48·7%)
Smoker	312 302 (23·4%)	162 076 (22·8%)	150 226 (24·2%)	595 547 (19·4%)	312 302/907 849 (34·4%)
Non-smoker	510 325 (38·3%)	281 309 (39·5%)	229 016 (36·9%)	1 167 424 (37·9%)	510 325/1 677 749 (30·4%)
Unknown	232 126 (17·4%)	145 220 (20·4%)	86 906 (14·0%)	1 022 833 (33·2%)	232 126/1 254 959 (18·5%)
**Blood pressure (systolic/diastolic)**					
Very high (>160/100 mm Hg)	39 468 (3·0%)	16 872 (2·4%)	22 596 (3·6%)	44 656 (1·5%)	39 468/84 124 (46·9%)
High (141–160/91–100 mm Hg)	182 220 (13·7%)	80 785 (11·3%)	101 435 (16·4%)	216 560 (7·0%)	182 220/398 780 (45·7%)
Normal (110–140/65–90 mm Hg)	855 123 (64·2%)	452 231 (63·5%)	402 892 (65·0%)	1 497 236 (48·6%)	855 123/2 352 359 (36·4%)
Low (<110/65 mm Hg)	13 006 (1·0%)	7130 (1·0%)	5876 (0·9%)	40 673 (1·3%)	13 006/53 679 (24·2%)
Unknown	158 199 (11·9%)	98 989 (13·9%)	59 210 (9·5%)	673 071 (21·9%)	158 199/831 270 (19·0%)
No investigation	83 977 (6·3%)	55 832 (7·8%)	28 145 (4·5%)	605 400 (19·7%)	83 977/689 377 (12·2%)

Data are n (%) or n/N (%).

* 1 indicates most deprived, 5 indicates least deprived.

† 1 indicates a large urban area, 6 indicates a remote rural area.

‡ Stage 3 chronic kidney disease refers to an estimated glomerular filtration rate of 30–59 mL per min.

**Figure 2:**
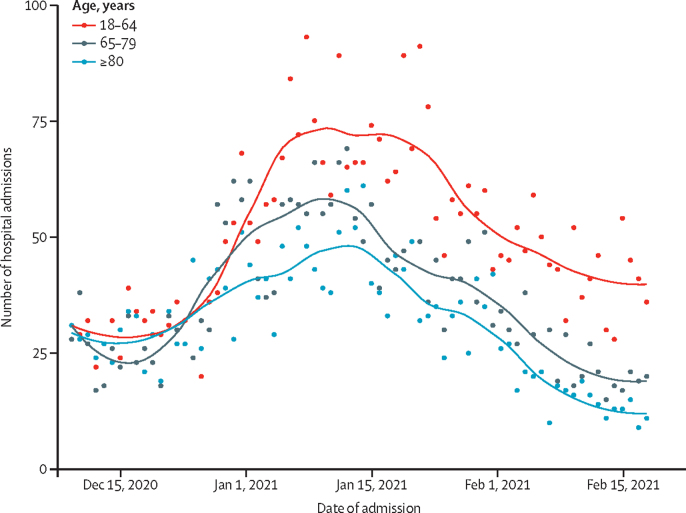
COVID-19 hospital admissions over time by age group

**Figure 3:**
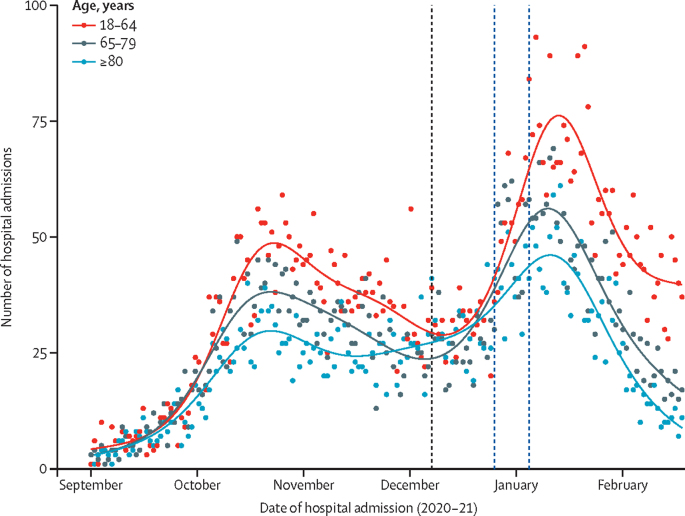
COVID-19 hospital admissions by age group from September, 2020, to February, 2021 The black dotted vertical line represents the start of vaccination (Dec 8, 2020) and the blue dotted lines represent the two lockdowns on Dec 26, 2020, and Jan 5, 2021. The smooth lines are obtained from fitting a generalised additive Poisson model to the admissions.

There was an overall downturn in the number of COVID-19 admissions to hospital over the study period (figure 2). This overall effect was not due to the vaccination programme because a downward trend was observed before mass vaccination. However, by use of an over-dispersed Poisson regression model, we note that hospital admissions declined more markedly in those aged 80 years and older, who were the first to be vaccinated and of whom 50% had been vaccinated by the end of the third week in January, 2021, compared with those aged 18–64 years, and that rate of decline accelerated in those aged 80 years and older (figure 3; appendix pp 13–14).

We found that vaccine effect at 28–34 days post-vaccination against COVID-19 hospital admissions among those receiving the first dose of the vaccine BNT162b2 was 91% (95% CI 85–94) and for ChAdOx1 was 88% (75–94; table 2).

**Table 2:** COVID-19 hospital admissions and days post-vaccination by vaccination status and type

	**Person-years**	**Number of events**	Age-adjusted RRs (95% CI)*	**Full-adjusted RRs (95% CI)**†	**Full and inverse propensity weighting adjusted RRs (95% CI)**‡	**Vaccine programme effect (95% CI)**
**Vaccinated overall**						
Unvaccinated	831 226	7698	1 (ref)	1 (ref)	1 (ref)	0
Vaccine dose 1 (0–6 days)§	19 917	156	0·43 (0·37 to 0·50)	0·38 (0·32 to 0·44)	0·25 (0·21 to 0·29)	75% (71 to 79)
Vaccine dose 1 (7–13 days)§	19 690	258	0·67 (0·59 to 0·76)	0·63 (0·56 to 0·72)	0·46 (0·41 to 0·52)	54% (48 to 59)
Vaccine dose 1 (14–20 days)	13 426	154	0·54 (0·46 to 0·63)	0·51 (0·43 to 0·60)	0·33 (0·28 to 0·39)	67% (61 to 72)
Vaccine dose 1 (21–27 days)	9119	72	0·37 (0·29 to 0·46)	0·33 (0·26 to 0·42)	0·24 (0·19 to 0·30)	76% (70 to 81)
Vaccine dose 1 (28–34 days)	6252	30	0·25 (0·17 to 0·36)	0·21 (0·15 to 0·30)	0·11 (0·08 to 0·17)	89% (83 to 92)
Vaccine dose 1 (35–41 days)	3822	21	0·36 (0·23 to 0·55)	0·23 (0·15 to 0·36)	0·22 (0·15 to 0·32)	78% (68 to 85)
Vaccine dose 1 (42+ days)	6047	32	0·51 (0·36 to 0·72)	0·28 (0·19 to 0·40)	0·26 (0·19 to 0·35)	74% (65 to 81)
**BNT162b2 (Pfizer–BioNTech)**						
Unvaccinated	734 031	6671	1 (ref)	1 (ref)	1 (ref)	0
Vaccine dose 1 (0–6 days)§	10 517	34	0·18 (0·13 to 0·25)	0·15 (0·10 to 0·21)	0·14 (0·10 to 0·19)	86% (81 to 90)
Vaccine dose 1 (7–13 days)§	10 991	119	0·57 (0·48 to 0·69)	0·42 (0·35 to 0·51)	0·47 (0·41 to 0·55)	53% (45 to 59)
Vaccine dose 1 (14–20 days)	7684	71	0·51 (0·40 to 0·64)	0·31 (0·24 to 0·39)	0·31 (0·25 to 0·38)	69% (62 to 75)
Vaccine dose 1 (21–27 days)	5672	38	0·41 (0·30 to 0·57)	0·21 (0·15 to 0·29)	0·22 (0·17 to 0·29)	78% (71 to 83)
Vaccine dose 1 (28–34 days)	4585	19	0·26 (0·16 to 0·40)	0·13 (0·08 to 0·21)	0·09 (0·06 to 0·15)	91% (85 to 94)
Vaccine dose 1 (35–41 days)	3292	20	0·40 (0·26 to 0·62)	0·18 (0·11 to 0·28)	0·22 (0·15 to 0·31)	78% (69 to 85)
Vaccine dose 1 (42+ days)	5996	31	0·38 (0·26 to 0·54)	0·20 (0·14 to 0·28)	0·23 (0·17 to 0·32)	77% (68 to 83)
**ChAdOx1 (Oxford–AstraZeneca)**						
Unvaccinated	743 142	7252	1 (ref)	1 (ref)	1 (ref)	0
Vaccine dose 1 (0–6 days)§	9222	122	0·46 (0·38 to 0·55)	0·43 (0·35 to 0·51)	0·28 (0·23 to 0·34)	72% (66 to 77)
Vaccine dose 1 (7–13 days)§	8699	139	0·48 (0·41 to 0·57)	0·53 (0·44 to 0·63)	0·32 (0·27 to 0·39)	68% (61 to 73)
Vaccine dose 1 (14–20 days)	5742	83	0·38 (0·30 to 0·47)	0·47 (0·37 to 0·58)	0·27 (0·21 to 0·34)	73% (66 to 79)
Vaccine dose 1 (21–27 days)	3447	34	0·23 (0·16 to 0·32)	0·31 (0·22 to 0·44)	0·19 (0·13 to 0·28)	81% (72 to 87)
Vaccine dose 1 (28–34 days)	1666	11	0·15 (0·08 to 0·26)	0·21 (0·12 to 0·39)	0·12 (0·06 to 0·25)	88% (75 to 94)
Vaccine dose 1 (35–41 days)	530	≤5	0·04 (0·01 to 0·29)	0·06 (0·01 to 0·44)	0·03 (0·00 to 0·37)	97% (63 to 100)
Vaccine dose 1 (42+ days)	51	≤5	0·44 (0·06 to 3·10)	0·68 (0·10 to 4·87)	0·41 (0·04 to 3·96)	59% (−296 to 96)

Poisson regression was used to provide all adjusted estimates. Individuals who had previously tested positive were excluded. RR=rate ratios. NA=not applicable. rtPCR=real-time reverse-transcription-PCR.

* Adjusted for age.

† Adjusted for time (in weeks), age, sex, Scottish Index of Multiple Deprivation, number of rtPCR tests before vaccination, and number of underlying medical conditions.

‡ Adjusted for time (in weeks), age, sex, Scottish Index of Multiple Deprivation, number of rtPCR tests before vaccination, number of underlying medical conditions, and inverse propensity of being vaccinated.

§ Any effects observed in less than 14 days are mainly due to vaccine programme effects.

Similar findings were observed in a pooled analysis for both vaccines against COVID-19 hospital admissions stratified by age group (table 3). At 28–34 days after vaccination, the vaccine effect estimate for those aged 18–64 years was 92% (95% CI 82–97) and for those aged 65–79 years was 93% (73–98). Vaccine effect for those aged 80 years and older at 28–34 days was 83% (95% CI 72–89).

**Table 3:** COVID-19 hospital admissions and days post-vaccination by age group and vaccination status and type

		**Person-years**	**Number of events**	Age-adjusted RRs (95% CI)*	**Full-adjusted RRs (95% CI)**†	**Full and inverse propensity weighting adjusted RRs (95% CI)**‡	**Vaccine programme effect (95% CI)**
**Vaccinated overall**							
18–64 years							
	Unvaccinated	66 1 060	3442	1 (ref)	1 (ref)	1 (ref)	0
	Vaccine dose 1 (0–6 days)§	6720	18	0·40 (0·25 to 0·64)	0·28 (0·18 to 0·46)	0·38 (0·29 to 0·50)	62% (50 to 71)
	Vaccine dose 1 (7–13 days)§	6667	55	1·29 (0·99 to 1·69)	0·88 (0·66 to 1·16)	1·10 (0·93 to 1·31)	−10% (−31 to 7)
	Vaccine dose 1 (14–20 days)	5458	25	0·77 (0·52 to 1·14)	0·49 (0·33 to 0·74)	0·58 (0·45 to 0·75)	42% (25 to 55)
	Vaccine dose 1 (21–27 days)	4769	12	0·43 (0·24 to 0·76)	0·27 (0·15 to 0·49)	0·38 (0·27 to 0·54)	62% (46 to 73)
	Vaccine dose 1 (28–34 days)	3859	≤5	0·13 (0·04 to 0·41)	0·09 (0·03 to 0·27)	0·08 (0·03 to 0·18)	92% (82 to 97)
	Vaccine dose 1 (35–41 days)	2797	7	0·43 (0·20 to 0·90)	0·26 (0·12 to 0·56)	0·40 (0·25 to 0·62)	60% (38 to 75)
	Vaccine dose 1 (42+ days)	5166	11	0·37 (0·20 to 0·66)	0·24 (0·13 to 0·45)	0·35 (0·24 to 0·51)	65% (49 to 76)
65–79 years							
	Unvaccinated	132 254	2449	1 (ref)	1 (ref)	1 (ref)	0
	Vaccine dose 1 (0–6 days)§	9598	42	0·23 (0·17 to 0·31)	0·26 (0·19 to 0·35)	0·12 (0·08 to 0·17)	88% (83 to 92)
	Vaccine dose 1 (7–13 days)§	9148	75	0·44 (0·35 to 0·55)	0·64 (0·50 to 0·82)	0·26 (0·20 to 0·35)	74% (65 to 80)
	Vaccine dose 1 (14–20 days)	4219	40	0·46 (0·34 to 0·63)	0·60 (0·43 to 0·83)	0·25 (0·17 to 0·36)	75% (64 to 83)
	Vaccine dose 1 (21–27 days)	1179	15	0·56 (0·34 to 0·94)	0·47 (0·28 to 0·79)	0·21 (0·11 to 0·39)	79% (61 to 89)
	Vaccine dose 1 (28–34 days)	498	≤5	0·28 (0·09 to 0·86)	0·16 (0·05 to 0·50)	0·07 (0·02 to 0·27)	93% (73 to 98)
	Vaccine dose 1 (35–41 days)	242	5	1·03 (0·43 to 2·48)	0·36 (0·14 to 0·91)	0·16 (0·05 to 0·49)	84% (51 to 95)
	Vaccine dose 1 (42+ days)	334	11	1·82 (1·00 to 3·28)	0·62 (0·34 to 1·15)	0·28 (0·13 to 0·58)	72% (42 to 87)
≥80 years							
	Unvaccinated	38 439	1807	1 (ref)	1 (ref)	1 (ref)	0
	Vaccine dose 1 (0–6 days)§	3072	96	0·68 (0·55 to 0·83)	0·44 (0·36 to 0·55)	0·26 (0·20 to 0·32)	74% (68 to 80)
	Vaccine dose 1 (7–13 days)§	3876	128	0·73 (0·61 to 0·88)	0·63 (0·52 to 0·77)	0·34 (0·27 to 0·41)	66% (59 to 73)
	Vaccine dose 1 (14–20 days)	3749	89	0·53 (0·43 to 0·65)	0·54 (0·43 to 0·68)	0·31 (0·24 to 0·39)	69% (61 to 76)
	Vaccine dose 1 (21–27 days)	3172	45	0·32 (0·23 to 0·42)	0·35 (0·26 to 0·49)	0·21 (0·15 to 0·30)	79% (70 to 85)
	Vaccine dose 1 (28–34 days)	1894	24	0·28 (0·18 to 0·41)	0·30 (0·19 to 0·46)	0·17 (0·11 to 0·28)	83% (72 to 89)
	Vaccine dose 1 (35–41 days)	782	9	0·24 (0·13 to 0·47)	0·21 (0·11 to 0·41)	0·13 (0·06 to 0·27)	87% (73 to 94)
	Vaccine dose 1 (42+ days)	548	10	0·37 (0·20 to 0·69)	0·24 (0·13 to 0·46)	0·15 (0·07 to 0·32)	85% (68 to 93)
**BNT162b2 (Pfizer–BioNTech)**							
18–64 years							
	Unvaccinated	642 027	3277	1 (ref)	1 (ref)	1 (ref)	0
	Vaccine dose 1 (0–6 days)§	5157	9	0·28 (0·14 to 0·53)	0·18 (0·09 to 0·36)	0·22 (0·15 to 0·34)	78% (66 to 85)
	Vaccine dose 1 (7–13 days)§	5757	46	1·30 (0·97 to 1·73)	0·84 (0·62 to 1·14)	1·06 (0·88 to 1·27)	−6% (−27 to 12)
	Vaccine dose 1 (14–20 days)	5141	24	0·79 (0·53 to 1·19)	0·49 (0·32 to 0·74)	0·58 (0·44 to 0·75)	42% (25 to 56)
	Vaccine dose 1 (21–27 days)	4663	12	0·44 (0·25 to 0·78)	0·27 (0·15 to 0·48)	0·37 (0·26 to 0·53)	63% (47 to 74)
	Vaccine dose 1 (28–34 days)	3804	≤5	0·14 (0·04 to 0·42)	0·08 (0·03 to 0·27)	0·08 (0·03 to 0·18)	92% (82 to 97)
	Vaccine dose 1 (35–41 days)	2773	7	0·43 (0·21 to 0·91)	0·25 (0·11 to 0·54)	0·37 (0·24 to 0·59)	63% (41 to 76)
	Vaccine dose 1 (42+ days)	5161	11	0·37 (0·20 to 0·67)	0·23 (0·12 to 0·42)	0·32 (0·22 to 0·47)	68% (53 to 78)
65–79 years							
	Unvaccinated	76 418	1994	1 (ref)	1 (ref)	1 (ref)	0
	Vaccine dose 1 (0–6 days)§	4487	13	0·11 (0·07 to 0·19)	0·12 (0·07 to 0·20)	0·07 (0·03 to 0·13)	93% (87 to 97)
	Vaccine dose 1 (7–13 days)§	4580	39	0·33 (0·24 to 0·46)	0·42 (0·30 to 0·58)	0·23 (0·15 to 0·34)	77% (66 to 85)
	Vaccine dose 1 (14–20 days)	1904	21	0·38 (0·25 to 0·59)	0·36 (0·23 to 0·56)	0·20 (0·12 to 0·34)	80% (66 to 88)
	Vaccine dose 1 (21–27 days)	399	5	0·40 (0·17 to 0·97)	0·14 (0·06 to 0·36)	0·09 (0·03 to 0·27)	91% (73 to 97)
	Vaccine dose 1 (28–34 days)	278	≤5	0·36 (0·11 to 1·10)	0·12 (0·04 to 0·36)	0·07 (0·02 to 0·27)	93% (73 to 98)
	Vaccine dose 1 (35–41 days)	188	5	0·91 (0·38 to 2·20)	0·23 (0·09 to 0·58)	0·14 (0·05 to 0·43)	86% (57 to 95)
	Vaccine dose 1 (42+ days)	329	11	1·20 (0·66 to 2·17)	0·38 (0·21 to 0·71)	0·24 (0·12 to 0·50)	76% (50 to 88)
≥80 years							
	Unvaccinated	15 963	1400	1 (ref)	1 (ref)	1 (ref)	0
	Vaccine dose 1 (0–6 days)§	497	12	0·27 (0·16 to 0·48)	0·14 (0·08 to 0·24)	0·11 (0·06 to 0·21)	89% (79 to 94)
	Vaccine dose 1 (7–13 days)§	654	34	0·60 (0·43 to 0·85)	0·28 (0·20 to 0·40)	0·18 (0·12 to 0·27)	82% (73 to 88)
	Vaccine dose 1 (14–20 days)	640	26	0·47 (0·32 to 0·70)	0·25 (0·17 to 0·37)	0·15 (0·10 to 0·24)	85% (76 to 90)
	Vaccine dose 1 (21–27 days)	611	21	0·40 (0·26 to 0·62)	0·23 (0·15 to 0·36)	0·15 (0·09 to 0·25)	85% (75 to 91)
	Vaccine dose 1 (28–34 days)	503	13	0·30 (0·17 to 0·52)	0·19 (0·11 to 0·34)	0·12 (0·06 to 0·24)	88% (76 to 94)
	Vaccine dose 1 (35–41 days)	331	8	0·28 (0·14 to 0·56)	0·18 (0·09 to 0·38)	0·13 (0·06 to 0·30)	87% (70 to 94)
	Vaccine dose 1 (42+ days)	506	9	0·21 (0·11 to 0·40)	0·22 (0·11 to 0·43)	0·15 (0·07 to 0·34)	85% (66 to 93)
**ChAdOx1 (Oxford–AstraZeneca)**							
18–64 years							
	Unvaccinated	626 849	3293	1 (ref)	1 (ref)	1 (ref)	0
	Vaccine dose 1 (0–6 days)§	1473	9	0·72 (0·37 to 1·39)	0·52 (0·26 to 1·02)	0·82 (0·56 to 1·20)	18% (−20 to 44)
	Vaccine dose 1 (7–13 days)§	910	9	1·20 (0·62 to 2·30)	0·81 (0·41 to 1·58)	0·99 (0·64 to 1·52)	1% (−52 to 36)
	Vaccine dose 1 (14–20 days)	317	≤5	0·40 (0·06 to 2·87)	0·28 (0·04 to 2·03)	0·25 (0·06 to 1·06)	75% (−6 to 94)
	Vaccine dose 1 (21–27 days)	105	0	0·00 (0·00 to NA)	0·00 (0·00 to NA)	0·00 (0·00 to NA)	100% (NA to 100)
	Vaccine dose 1 (28–34 days)	55	0	0·00 (0·00 to NA)	0·00 (0·00 to NA)	0·00 (0·00 to NA)	100% (NA to 100)
	Vaccine dose 1 (35–41 days)	24	0	0·00 (0·00 to NA)	0·00 (0·00 to NA)	0·00 (0·00 to NA)	100% (NA to 100)
	Vaccine dose 1 (42+ days)	5	0	0·00 (0·00 to NA)	0·00 (0·00 to NA)	0·00 (0·00 to NA)	100% (NA to 100)
65–79 years							
	Unvaccinated	81 523	2194	1 (ref)	1 (ref)	1 (ref)	0
	Vaccine dose 1 (0–6 days)§	4761	29	0·22 (0·15 to 0·32)	0·24 (0·16 to 0·35)	0·12 (0·08 to 0·19)	88% (81 to 92)
	Vaccine dose 1 (7–13 days)§	4567	36	0·28 (0·20 to 0·39)	0·45 (0·31 to 0·63)	0·21 (0·14 to 0·31)	79% (69 to 86)
	Vaccine dose 1 (14–20 days)	2316	19	0·28 (0·18 to 0·44)	0·46 (0·29 to 0·74)	0·21 (0·12 to 0·37)	79% (63 to 88)
	Vaccine dose 1 (21–27 days)	780	10	0·42 (0·22 to 0·78)	0·69 (0·37 to 1·32)	0·32 (0·15 to 0·69)	68% (31 to 85)
	Vaccine dose 1 (28–34 days)	220	0	0·00 (0·00 to NA)	0·00 (0·00 to NA)	0·00 (0·00 to NA)	100% (NA to 100)
	Vaccine dose 1 (35–41 days)	55	0	0·00 (0·00 to NA)	0·00 (0·00 to NA)	0·00 (0·00 to NA)	100% (NA to 100)
	Vaccine dose 1 (42+ days)	5	0	0·00 (0·00 to NA)	0·00 (0·00 to NA)	0·00 (0·00 to NA)	100% (NA to 100)
≥80 years							
	Unvaccinated	35 266	1765	1 (ref)	1 (ref)	1 (ref)	0
	Vaccine dose 1 (0–6 days)§	2493	84	0·69 (0·56 to 0·86)	0·50 (0·40 to 0·63)	0·28 (0·22 to 0·36)	72% (64 to 78)
	Vaccine dose 1 (7–13 days)§	3221	94	0·61 (0·50 to 0·75)	0·64 (0·51 to 0·81)	0·37 (0·29 to 0·47)	63% (53 to 71)
	Vaccine dose 1 (14–20 days)	3109	63	0·43 (0·33 to 0·55)	0·56 (0·42 to 0·74)	0·37 (0·28 to 0·50)	63% (50 to 72)
	Vaccine dose 1 (21–27 days)	2561	24	0·20 (0·13 to 0·29)	0·30 (0·19 to 0·47)	0·23 (0·14 to 0·37)	77% (63 to 86)
	Vaccine dose 1 (28–34 days)	1391	11	0·16 (0·09 to 0·30)	0·26 (0·14 to 0·50)	0·19 (0·09 to 0·40)	81% (60 to 91)
	Vaccine dose 1 (35–41 days)	451	≤5	0·04 (0·01 to 0·32)	0·07 (0·01 to 0·53)	0·05 (0·00 to 0·56)	95% (44 to 100)
	Vaccine dose 1 (42+ days)	42	≤5	0·48 (0·07 to 3·39)	0·84 (0·12 to 6·03)	0·64 (0·07 to 6·24)	36% (−524 to 93)

Poisson regression was used to provide all adjusted estimates. Individuals who had previously tested positive were excluded. RR=rate ratios. NA=not applicable. rtPCR=real-time reverse-transcription-PCR.

* Adjusted for age.

† Adjusted for time (in weeks), age, sex, Scottish Index of Multiple Deprivation, number of rtPCR tests before vaccination, and number of underlying medical conditions.

‡ Adjusted for time (in weeks), age, sex, Scottish Index of Multiple Deprivation, number of rtPCR tests before vaccination, number of underlying medical conditions, and inverse propensity of being vaccinated.

§ Any effects observed in less than 14 days are mainly due to vaccine programme effects.

Among those aged 80 years and older at 28–34 days post-vaccination, vaccine effect against COVID-19 hospital admission for those who received the first dose of the BNT162b2 mRNA vaccine was 88% (95% CI 76–94), and for ChAdOx1 was 81% (60–91; table 3).

The falsification analysis involving shifting the vaccination date back 2 months to a period when vaccination could not have had an effect showed no difference between the vaccinated and unvaccinated groups over the whole follow-up period (appendix pp 6–9).

## Discussion

This national prospective cohort study comprising almost the entire Scottish population found that the mass roll-out of the first doses of the BNT162b2 mRNA or ChAdOx1 vaccines was associated with protection against COVID-19 admission to hospital. A vaccine effect of 91% for the BNT162b2 mRNA vaccine and 88% for the ChAdOx1 vaccine were found against COVID-19 hospital admissions at 28–34 days after vaccination. In the oldest age group (≥80 years), on the basis of a pooled analysis for both vaccines, we observed a vaccine effect of 83% against COVID-19 hospital admissions at this same timepoint. For the same age group and at the same time, a vaccine effect of 88% was found for the BNT162b2 mRNA vaccine, and 81% for the ChAdOx1 vaccine.

A study from Israel (Clalit Health Services) has reported on the vaccine effect of BNT162b2 mRNA. An analysis of data on more than 1·1 million individuals showed that after the first dose of vaccination, an effect of 74% was found 14–20 days after immunisation and an effect of 78% was found 21–27 days after immunisation against SARS-CoV-2 hospital admission.^15^ Public Health England has reported that single dose vaccine effects against hospital admission for BNT162b2 mRNA and ChAdOx1 were 80%.^16^ Complementary to these studies, we found a similar vaccine effect against COVID-19 hospital admission for the BNT162b2 mRNA and ChAdOx1 vaccines after a single dose.

This is, to our knowledge, the first national population-level study assessing the programme effect of currently licensed COVID-19 vaccines on a severe COVID-19 outcome. Our study has several strengths. We developed a national linked dataset and have created a platform that allowed rapid access to and analysis of data on vaccination status and medical condition status from routinely collected electronic health record data and national databases.^8^, ^17^ This study is therefore less susceptible to recall or misclassification bias than are studies of samples of the population. The use of a large population aided study power, facilitating estimation of vaccine effect in multiple age groups and time intervals after the first dose of the vaccination. We are likely to have excellent generalisability across the UK and potentially across other countries with similar national vaccination programmes using these same vaccines.

Our study also had several limitations. First, we estimated vaccine effect against COVID-19 hospital admission. However, there are other outcomes of interest, including general practice and accident and emergency department consultations, intensive care unit admissions, deaths, and rate of secondary SARS-CoV-2 infections within households, in addition to maternal and neonatal outcomes. We did not estimate vaccine effect against these outcomes. Second, although our vaccine effect estimates were adjusted for potential confounders and despite the reassurance provided by the falsification of exposure sensitivity analysis, unmeasured confounders could still have influenced our estimates. Furthermore, given the limited period of follow-up, we did not attempt to identify a peak vaccine effect estimate for each vaccine. Finally, we were unable to compare vaccine effects between the two vaccines; this is primarily because of the non-experimental design of our study and because the target population differed between vaccines in this initial roll-out period.

Additional non-vaccine effects on hospital admissions could have resulted in potential bias. These included new lockdowns and changes from tier restrictions to full lockdowns across Scotland. However, partial lockdowns were present before the start of the vaccination programme and a full lockdown for the entire population—including those vaccinated—began on Dec 26, 2020, and was reinforced on Jan 5, 2021. Overall, data available from UK serial surveys to the Scottish Government and Public Health Scotland suggested good compliance with these partial and full lockdowns,^18^ which are unlikely to have been operating differentially between vaccinated and unvaccinated people with the exception of health-care workers and social-care workers because they were considered essential workers. We adjusted for time to adjust for any impact on the effect of these interventions and the course of the pandemic on our estimates of vaccine effects. Other limitations of our study include the ChAdOx1 vaccine being predominantly used in older people and available only from Jan 4, 2021, giving less time for follow-up. Finally, although we had large population samples, an insufficient number of people had received the second dose of the vaccines to reliably study vaccine effect after receiving a full course of vaccination. However, the vaccine effect of a single dose is of policy interest when considering the ongoing debate over whether to defer a second dose to allow more rapid population coverage.

There are likely to be mechanisms that have contributed mainly to the early (<14 days) effects we observed in most age groups. We believe that these effects might be due to the way in which the Scottish vaccination programme was conceptualised and operationalised. Notably, individuals who were prioritised for vaccination received a written invitation to attend for a scheduled appointment about 14 days before the vaccination date. However, they were asked to reschedule their appointment if they had recently (within the past 4 weeks) had symptoms suggestive of COVID-19, a positive COVID-19 test, or were self-isolating. They also received written advice emphasising the importance of behavioural measures to minimise the risk of contracting the SARS-CoV-2 virus. Furthermore, at the time of the vaccination, additional checks were made to ensure that they had not recently had suggested or confirmed COVID-19 and the behavioural advice was reinforced both verbally and with an additional leaflet. Possible explanations for these early effects therefore include: (1) a time-limited healthy vaccinated bias arising from the instruction that those who recently had COVID-19, tested positive, or were self-isolating were asked to defer vaccination;^19^ (2) reinforcement of the importance of following behavioural advice before and during vaccination;^19^, ^20^ (3) early responses in those who were not SARS-CoV-2 naive (>10% of the Scottish population);^21^, ^22^ and (4) a time-independent healthy vaccinated bias in which vaccines might not have been offered to or were declined by the most frail.^23^ We attempted to adjust for the time-independent healthy vaccinated effect using a functional variable, namely dementia.^14^ We had hoped to use additional functional variables such as nursing home residency and need for home help, but these variables were not available to us.

By contrast, the later observed effects (>14 days) are much more likely to be mainly driven by traditional vaccine effects because the impact of the vaccine programme effects are likely to have waned by this timepoint. However, our main timepoint of interest, namely 28–34 days, probably reflects the true effects of the vaccine.

Monitoring the effect of currently licensed vaccines in the general population needs to be continued in Scotland and the other UK nations, especially in high-risk subgroups such as those in care homes where more data will be needed to produce reliable vaccine effect estimates. Similarly, further monitoring is required to assess the effect of receiving two doses rather than one dose. Robust observational epidemiological studies should be carried out to measure the coverage of these newly introduced vaccines in relation to demographic and other population characteristics and to detect adverse events. These post-marketing observational studies will add value to the pre-licensure clinical trials as they can assess so-called real-life effects of the COVID-19 vaccines and the effect of the vaccination programme at a population level. We plan in due course to report on the effectiveness of the first dose on mortality and adverse events associated with the first dose. We also plan to report on any possible waning of the first-dose effects, on post-vaccine first-dose COVID-19 hospital admissions and deaths, and on second-dose effects.

In summary, we provide national evidence that the roll-out of the first doses of the BNT162b2 mRNA and ChAdOx1 vaccines were associated with reductions in hospital admissions due to COVID-19 in high-risk adults in Scotland.

## Data sharing

A data dictionary covering the datasets used in this study can be found at https://github.com/EAVE-II/EAVE-II-data-dictionary. All code used in this study is publicly available at https://github.com/EAVE-II/Covid-VE. The data used in this study are sensitive and will not be made publicly available.

## Declaration of interests

AS and JMcM are members of the Scottish Government chief medical officer's COVID-19 Advisory Group. JMcM is a member of the New and Emerging Respiratory Virus Threats Advisory Group (NERVTAG) and AS is a member of the NERVTAG Risk Stratification Subgroup. JMcM is a member of the Scientific Advisory Group on Emergencies (SAGE) and chairs the COVID Scottish National Incident Management Team and the Scientific Committee of the European Centre for Disease Prevention and Control-funded and WHO-funded IMOVE-COVID-19 group. CRS declares funding from the Medical Research Council, the National Institute of Health Research, Chief Scientist Office, and New Zealand Ministry for Business, Innovation and Employment and Health Research Council during the conduct of this study. SVK is co-chair of the Scottish Government's Expert Reference Group on COVID-19 and ethnicity; is a member of the SAGE subgroup on ethnicity; and acknowledges funding from a National Health Service Research Scotland Senior Clinical Fellowship, the Medical Research Council, and Chief Scientist Office. CR is a member of the Scottish Government chief medical officer's COVID-19 Advisory Group, Scientific Pandemic Influenza Group on Modelling, and Medicines and Healthcare products Regulatory Agency Vaccine Benefit and Risk Working Group. DB reports employment by Queen's University Belfast, Public Health Agency, and a secondment to Northern Ireland's Department of Health. SdL reports that he is the director of the Oxford-Royal College of General Practitioners Research and Surveillance Centre and that its principal funder is Public Health England. SdL has received funding through the University of Oxford for studies from AstraZeneca, Daiichi Sankyo, Eli Lilly, Sanofi, GSK, Merck Sharp & Dohme, Seqirus, and Takeda; and has been a member of advisory boards for influenza for Seqirus and Sanofi. FDRH reports that the University of Oxford received research funding from Her Majesty's Government for the ChadOx1 vaccine trial programme and entered a partnership with AstraZeneca for the manufacture and distribution of the vaccine. FDRH was not involved in these relationships. All other authors report no competing interests.
