# Supplementary material for: Interim findings from first-dose mass COVID-19 vaccination roll-out and COVID-19 hospital admissions in Scotland: a national prospective cohort study
Source: Lancet. 2021 May 1;397(10285):1646–57. doi: 10.1016/S0140-6736(21)00677-2 (PMC8064669; doi:10.1016/S0140-6736(21)00677-2)
Supplement: Supplementary appendix [file mmc1.pdf]

THE LANCET

Supplementary appendix

This appendix formed part of the original submission and has been peer reviewed. We post it as supplied by the authors.

Supplement to: Vasileiou E, Simpson CR, Shi T, et al. Interim findings from first-dose mass COVID-19 vaccination roll-out and COVID-19 hospital admissions in Scotland: a national prospective cohort study. *Lancet* 2021; published online April 23. http://dx.doi.org/10.1016/S0140-6736(21)00677-2.

Supplemental File S1: Context of vaccine roll-out in Scotland

Supplemental Table 1. Joint Committee on Vaccination and Immunisation (JCVI) COVID-19 vaccination priority list on 30 December 2020

Order of priority	Group*
1	Residents in a care home for older adults and their carers
2	All those 80 years of age and over and frontline health and social care workers
3	All those 75 years of age and over
4	All those 70 years of age and over and clinically extremely vulnerable individuals
5	All those 65 years of age and over
6	All individuals aged 16 years to 64 years with underlying health conditions which put them at higher risk of serious disease and mortality
7	All those 60 years of age and over
8	All those 55 years of age and over
9	All those 50 years of age and over

*These groups represent around 99% of preventable mortality from COVID-19

The vaccine roll-out strategy has been determined by an independent UK-wide body, namely the Joint Commission on Vaccinations and Immunisation (JCVI),[1] which has prioritised vaccinations to adults on the basis of assessing the risk of serious COVID-19 outcomes, in particular hospitalisations and deaths.[1]

Individuals in these priority groups received a written invitation ~14 days before their appointment. They were asked however to delay their vaccination if they had recently had COVID-19, tested positive or were self-isolating. These invitations were accompanied by written advice on the need to observe behavioural measures to reduce the risk of contracting the infection.

Prior to vaccination, checks were made by the trained administering staff to see if individuals had COVID-19 or tested positive in the preceding 4 weeks; if so, the vaccination was deferred. Immediately following vaccination, individuals received both verbal and written advice on the need to maintain behavioural measures, particularly in the 2-3 weeks following vaccination.

Because of the different storage requirements for the two vaccines, GPs have administered the Oxford-AstraZeneca vaccine and vaccine centres have mainly administered the Pfizer-BioNTech vaccine. Guided by JCVI priorities, GPs began by focusing their efforts on: a) the mobile elderly who they vaccinated in their general practice surgeries; and b) care home residents affiliated with general practices. Vaccination centres began with focusing on health and social care providers before extending to other JCVI priority groups. By February 22nd 2021, Group 7 vaccination was underway and a full roll-out of invitations to Group 6 (Supplemental Table 1) started.

[1] Joint Committee on Vaccination and Immunisation. Priority groups for coronavirus (COVID-19) vaccination: advice from the JCVI, 30 December 2020. Available from: https://www.gov.uk/government/publications/priority-groups-for-coronavirus-covid-19-vaccination-advice-from-the-jcvi-30-december-2020

Supplemental File S2: Linkage of datasets

Supplemental Figure 1. Data linkage diagram*

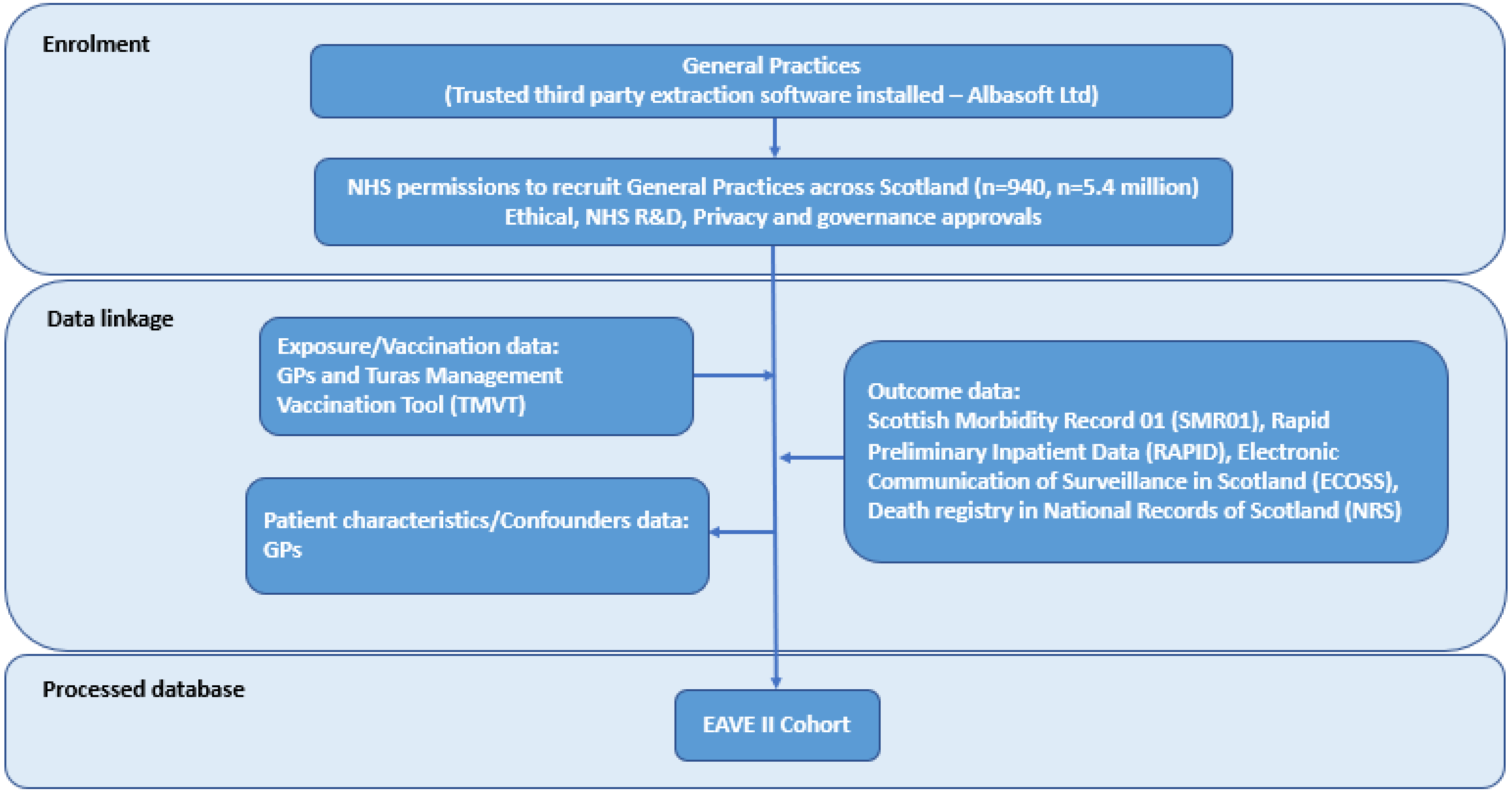

***Community Health Index (CHI) numbers were used to link all datasets. Details on these datasets are available in our published protocol (Simpson CR, Robertson C, Vasileiou E, *et al.* Early Pandemic Evaluation and Enhanced Surveillance of COVID-19 (EAVE II): protocol for an observational study using linked Scottish national data. *BMJ Open* 2020;**10:**e039097. doi: 10.1136/bmjopen-2020-039097)

There are two core methods of recording vaccine delivery, the national TMVT system and local GP IT systems. TMVT was developed as a web application by National Education Scotland (NES). It is in general the preferred method of recording a vaccination where this is done outside the normal vaccine locations, predominantly dedicated vaccination centres and community programmes. Most vaccinations delivered in general practice settings are recorded in local IT systems; there are a few geographical areas that have however mandated the use of TMVT in every setting, including GP practices. Currently GP's are paid per 100 vaccines administered so they are highly motivated to record information accurately. If this is not recorded to a minimum standard, they will not receive payment. All vaccines administered through vaccination centres and community programmes are accounted for on a daily basis. All vaccines recorded via TMVT are transferred to the national clinical datastore (NCDS) then to Albasoft on a daily basis. At 9pm each night, these are loaded into a secure database and each practice "polls" the data store as part of the ESCRO data pump run between 12:00am and 5:00am each day to request the records for their specific practice. These are then loaded into a local queue at the practice for processing later in the day. As part of the same data pump run, the local GP IT system is queried and all vaccination records for the previous day are extracted (with a 10 day overlap to catch any retrospective recording) These records are then transferred back to Albasoft and collated into a single data source which is returned to the National Clinical Data Store (NCDS) at 8am each morning. As a result, all vaccinations recorded either by TMVT or GP IT systems pass through Albasoft in a 24-hour cycle. As part of the agreement to provide these data for EAVE II, vaccination records from both the TMVT and GP IT systems are transferred each day following the National Clinical Data Store processing to the EAVE II secure datastore in Public Health Scotland (PHS). This ensures that the EAVE II data are as up to date as possible. It is therefore extremely unlikely that any vaccinations will have been missed.

Supplemental File S3: International Classification of Diseases (ICD)-10 codes

Supplemental Table 1. ICD-10 codes for COVID-19 illness

Code	Description
U07.1	COVID-19, virus identified
U07.2	COVID-19, virus not identified
Source: https://www.who.int/classifications/icd/COVID-19-coding-icd10.pdf	

Supplemental File S4: Predicted curved from Cox models for COVID-19 Hospitalisations and Falsification of exposure analysis

Supplemental Figure 1. Predicted curves, and 95% confidence limits, from a cox model adjusting for age, sex, deprivation (SIMD), number of tests and number of. comorbid conditions. Vaccinated from date of vaccination HR = 0.47 (95% CI 0.43 to 0.51). This shows that the vaccinated group diverges from the unvaccinated groups after 4 days.

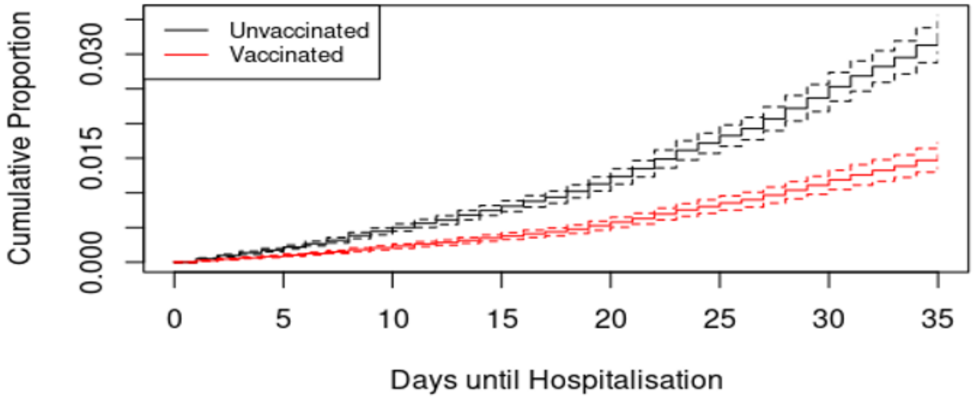

Supplemental Figure 2. Predicted curves from a cox model adjusting for age, sex, SIMD, number of. tests and number of. comorbid conditions with stratification on calendar period. Vaccinated from date of vaccination HR = 0.47 (95% CI 0.43 to 0.51). This shows that the vaccinated group diverges from the unvaccinated groups after vaccination. Calendar time is stratified into weeks from the beginning of the vaccination programme. This is necessary as the pattern of the epidemic changed over the observation period.

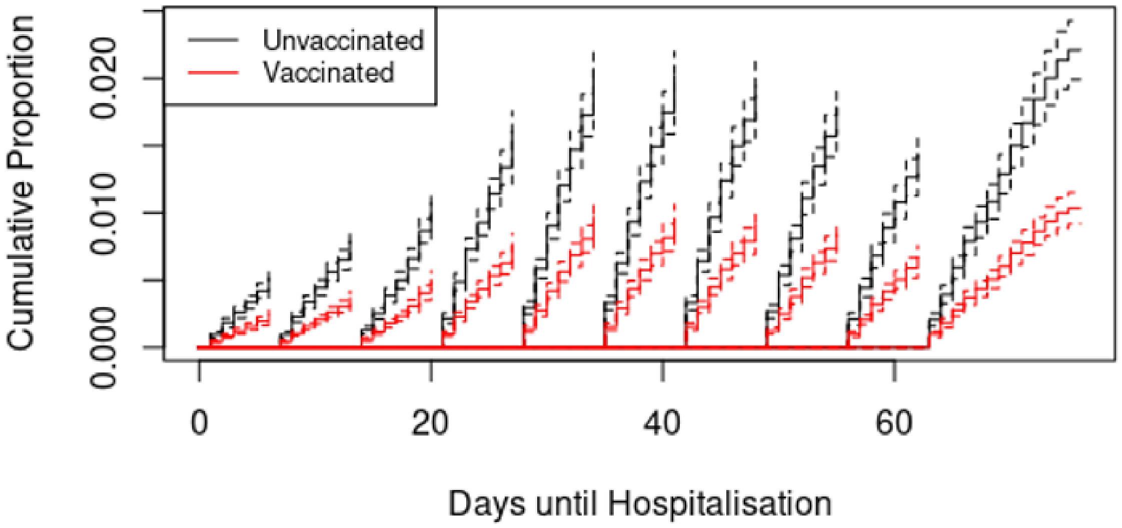
Unvaccinated
Vaccinated
Cumulative Proportion
0.000
0.010
0.020
0
20
40
60
Days until Hospitalisation

Supplemental Table 1. COVID-19 Hospitalisation moving Vaccination back two months (assuming 10% of dead participants were vaccinated). In this analysis we started the observation period on 8th October and followed up all cohort members until Dec 7th. The vaccinations from the period from Dec 8th onwards were moved back 2 months so that individuals had a pseudo vaccination at a time there was no intervention. One of the problems with this analysis is that individuals who died between 8th October and 7th December could not have been vaccinated and many of them would have had the covid hospitalisation endpoint before death. To compensate for this we assumed that 10% of those who died were vaccinated before death and sampled their vaccination date from the existing vaccination register. The results in the table show that there is no association between the pseudo vaccination and covid hospitalisation as all but one of the confidence intervals for the rate ratios span 1. There is no evidence of a reduced risk at 0-6 days. The simulation was repeated with a greater percentage vaccinated and that resulted in larger hazard ratios. The full covariate adjustment was not used in this investigation but the minimal adjustment included age, sex, deprivation and number of co morbid conditions. A Poisson generalised regression model was used to obtain the estimates

			Age adjusted			Minimal adjustment – No propensity score		
Vaccination status	Person years	Number of events	Rate Ratio (RR) (raw)	Lower 95% CI_(raw)	Upper 95% CI (raw)	RR (adjusted)	Lower 95% CI (adjusted)	Upper 95% CI (adjusted)
Unvaccinated	712858	5277	NA	NA	NA	NA	NA	NA
Vaccine dose 1 (0-6 days)	10024	137	0.87	0.73	1.03	0.91	0.76	1.08
Vaccine dose 1 (7-13 days)	8484	92	0.73	0.59	0.90	0.77	0.62	0.95
Vaccine dose 1 (14-20 days)	5708	71	0.98	0.77	1.24	1.03	0.81	1.31
Vaccine dose 1 (21-27 days)	3529	47	1.32	0.99	1.76	1.35	1.01	1.80

Vaccine dose 1 (28-34 days)	2204	21	1.08	0.71	1.66	1.05	0.68	1.61
Vaccine dose 1 (35-41 days)	1649	11	0.80	0.44	1.44	0.79	0.44	1.43
Vaccine dose 1 (42+ days)	1862	15	1.10	0.66	1.82	1.15	0.69	1.92
Vaccine dose 2 (0-6 days)	118	≤5	2.44	0.61	9.78	3.25	0.81	13.01
Vaccine dose 2 (7+ days)	240	0	0.00	0.00	Inf	0.00	0.00	Inf

Supplemental File S5: Baseline characteristics of ≥80 year-olds

Table 1. ≥80 years - Baseline characteristics by vaccine status and timing (BNT162b2 and ChAdOx1).

Variable	Level	Vaccinated 08-Dec to 03-Jan (%)	Vaccinated 03-Jan to 16-Jan (%)	Vaccinated 17-Jan to 31-Jan (%)	Vaccinated 01-Feb to 21 Feb (%)	Unvaccinated (%)
Mean age						
	NA	87.5	85.5	84.2	85.2	86.9
Sex						
	F	8374 (74.4%)	26908 (61.4%)	78697 (59.8%)	14563 (65.2%)	36413 (59.8%)
	M	2877 (25.6%)	16937 (38.6%)	52986 (40.2%)	7767 (34.8%)	24444 (40.2%)
Socio-economic Status						

	1 - High	1952 (17.3%)	6118 (14%)	20107 (15.3%)	3462 (15.5%)	11442 (18.8%)
	2	1869 (16.6%)	8174 (18.6%)	26705 (20.3%)	4768 (21.4%)	12035 (19.8%)
	3	2629 (23.4%)	9127 (20.8%)	28095 (21.3%)	4539 (20.3%)	12590 (20.7%)
	4	2495 (22.2%)	9781 (22.3%)	27431 (20.8%)	4523 (20.3%)	11983 (19.7%)
	5- Low	1818 (16.2%)	10424 (23.8%)	28865 (21.9%)	4947 (22.2%)	11009 (18.1%)
	NA	488 (4.3%)	221 (0.5%)	480 (0.4%)	91 (0.4%)	1798 (3%)
Number of comorbidities						
	0	813 (7.2%)	9029 (20.6%)	31207 (23.7%)	4008 (17.9%)	39447 (64.8%)
	1	1340 (11.9%)	10730 (24.5%)	34468 (26.2%)	5330 (23.9%)	6501 (10.7%)

	2	2285 (20.3%)	9761 (22.3%)	28829 (21.9%)	5064 (22.7%)	5688 (9.3%)
	3	2617 (23.3%)	6891 (15.7%)	18878 (14.3%)	3696 (16.6%)	4187 (6.9%)
	4	2011 (17.9%)	4112 (9.4%)	10358 (7.9%)	2267 (10.2%)	2614 (4.3%)
	5+	2185 (19.4%)	3322 (7.6%)	7943 (6%)	1965 (8.8%)	2420 (4%)

Individuals vaccinated in the first period 8th December 2020 to 3 January 2021 received BNT162b2, while individuals vaccinated in the later three periods could have received either but predominantly will have received ChAdOx1.

Supplemental File S6: Percentage and rate of hospital admissions over time by age group.

Figure 1. Percentage of hospital admissions over time by age group*
(The black dotted vertical line represents the start of vaccination (8 December 2020) and the dotted blue lines represent the two lockdowns on 26 December 2020 and 5 January 2021)

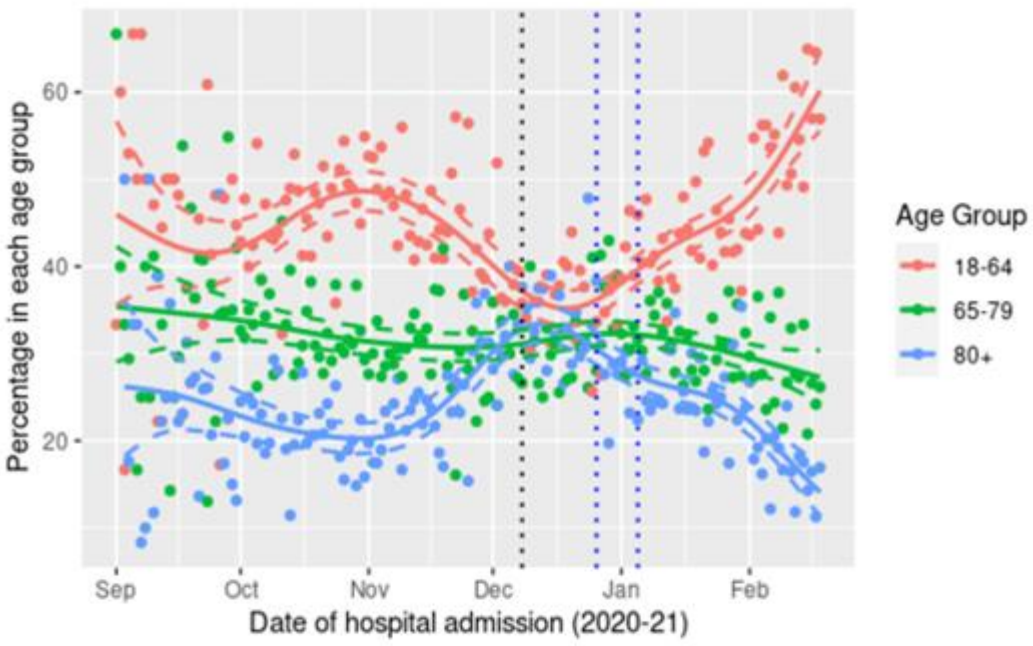

Figure 2. Rate of hospital admissions over time by age group*
(The black dotted vertical line represents the start of vaccination (8 December 2020) and the dotted blue lines represent the two lockdowns on 26 December 2020 and 5 January 2021)

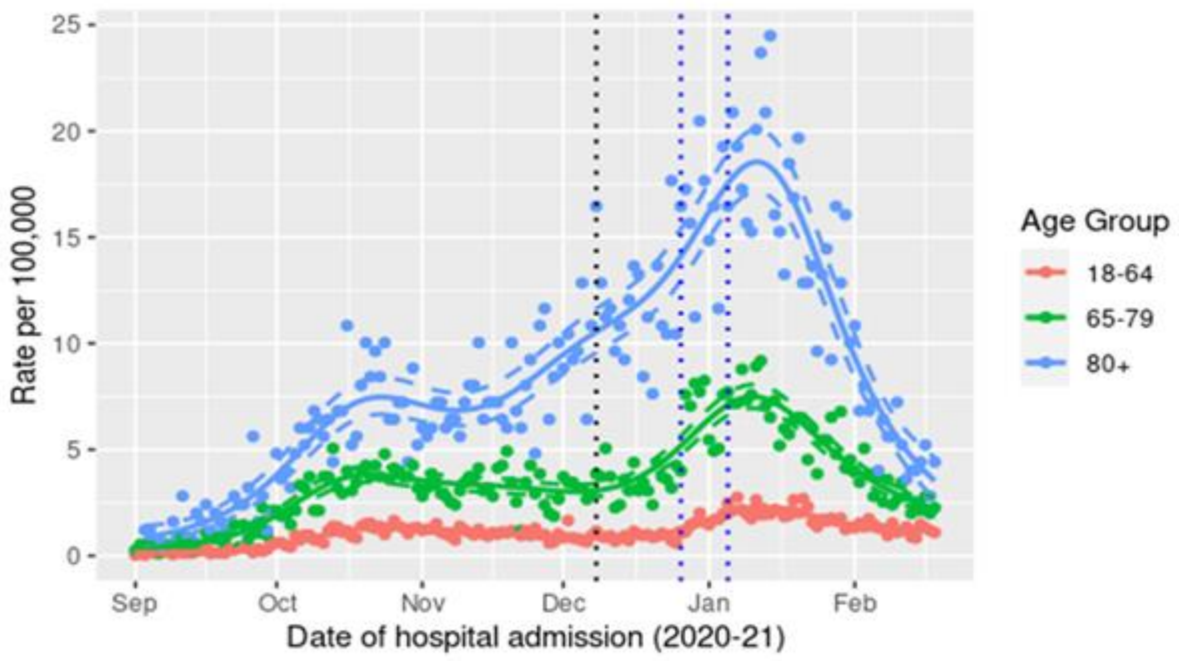

* There are a number of potential explanations for the changing trends Figures 1 and 2 which are unrelated to the vaccination programme. From mid-November the prevalence of the Kent B.1.1.7 variant increased in Scotland such that by the end of the observation period it was the dominant strain in Scotland. The relaxation of restrictions around the 11th December when many regions in the west of Scotland moved out of level 4 and the emergence of this strain, which affected all age groups in the community equally, is associated with the increase in hospitalised cases. It may also be associated with the increase in the proportion of younger adults among those admitted to hospital from mid-December onwards.

The turning over of the hospitalisation rates could not have been influenced by the vaccination programme. A national lockdown was introduced on the 26th December 2020 and strengthened with additional restrictions on 5th January 2021. In Scotland, the median time from testing to admission is 4 days with 75% admitted within 7 days of testing. Allowing time for infection to symptoms of 5-7 days suggests expecting an impact on hospitalisations within 14 days. Using the admission data from 9th January 2021 onwards, we find a greater reduction in the number of admissions per day among 80+ year olds of 3.7% (95% CI 3.1 to 4.3) per day compared to the 18-64 group where there was a 1.8% reduction per day (95%CI 1.4 to 2.2), with the 65-79 in the middle 3.1% (2.5 to 3.6). (P<0.0001 using an over-dispersed Poisson regression model).

Approximately 50% of the 80+ age group were vaccinated by the 24th January. Introducing a change point at 24th January 2021 for the three groups, we find that there is a change in slope for the 80+ group. Up to 24th January 2021, the reduction is 2.1% per day (95% CI 0.6 to 3.6) but after that date there is an additional reduction of 2.8% (95% CI 0.4, 5.1) per day. In the other two age groups, there was also a reduction in the slope post 24th January 2021, but this was lower at 1.1% in both groups with the confidence interval spanning 0. Furthermore, the change in the slopes was not the same in all 3 age groups (p=0.047).

Repeating this analysis by starting it on 2 January 2021 (i.e. one week after the lockdown) yields the same conclusions that the rate of decline in the hospitalisations was greater post-lockdown among the 80+ compared to the 18-64 year-olds and there is evidence of a change in slope after 24th January 2021 in the 80+ age group.

This post-hoc exploratory ecological analysis thus provides further supportive evidence that there was an impact of vaccination programme on hospitalisations.

Supplemental File S7: Reporting checklists

Supplemental Table 1. RECORD Checklist

	Item No.	STROBE items	Location in manuscript where items are reported	RECORD items	Location in manuscript where items are reported
**Title and abstract**					
	1	(a) Indicate the study's design with a commonly used term in the title or the abstract (b) Provide in the abstract an informative and balanced summary of what was done and what was found		RECORD 1.1: The type of data used should be specified in the title or abstract. When possible, the name of the databases used should be included.<br><br>RECORD 1.2: If applicable, the geographic region and timeframe within which the study took place should be reported in the title or abstract.<br><br>RECORD 1.3: If linkage between databases was conducted for the study, this should be clearly stated in the title or abstract.	p. 1, 4
**Introduction**					
Background rationale	2	Explain the scientific background and rationale for the investigation being reported			p. 7-8

Objectives	3	State specific objectives, including any prespecified hypotheses			p. 7-8
**Methods**					
Study Design	4	Present key elements of study design early in the paper			p. 8
Setting	5	Describe the setting, locations, and relevant dates, including periods of recruitment, exposure, follow-up, and data collection			p. 8
Participants	6	*(a) Cohort study* - Give the eligibility criteria, and the sources and methods of selection of participants. Describe methods of follow-up<br>*Case-control study* - Give the eligibility criteria, and the sources and methods of case ascertainment and control selection. Give the rationale for the choice of cases and controls<br>*Cross-sectional study* - Give the eligibility criteria, and the sources and methods of selection of participants		RECORD 6.1: The methods of study population selection (such as codes or algorithms used to identify subjects) should be listed in detail. If this is not possible, an explanation should be provided.<br><br>RECORD 6.2: Any validation studies of the codes or algorithms used to select the population should be referenced. If validation was conducted for this study and not published elsewhere, detailed methods and results should be provided.<br><br>RECORD 6.3: If the study involved linkage of databases, consider use of a flow diagram or other graphical display to demonstrate the data linkage process, including the number of individuals with linked data at each stage.	p. 8-9

		(b) Cohort study - For matched studies, give matching criteria and number of exposed and unexposed<br>*Case-control study* - For matched studies, give matching criteria and the number of controls per case			
Variables	7	Clearly define all outcomes, exposures, predictors, potential confounders, and effect modifiers. Give diagnostic criteria, if applicable.		RECORD 7.1: A complete list of codes and algorithms used to classify exposures, outcomes, confounders, and effect modifiers should be provided. If these cannot be reported, an explanation should be provided.	p. 8-9
Data sources/ measurement	8	For each variable of interest, give sources of data and details of methods of assessment (measurement).<br>Describe comparability of assessment methods if there is more than one group			p. 8-9
Bias	9	Describe any efforts to address potential sources of bias			p. 8-9
Study size	10	Explain how the study size was arrived at			N/A
Quantitative variables	11	Explain how quantitative variables were handled in			p. 9-11

		the analyses. If applicable, describe which groupings were chosen, and why			
Statistical methods	12	(a) Describe all statistical methods, including those used to control for confounding<br>(b) Describe any methods used to examine subgroups and interactions<br>(c) Explain how missing data were addressed<br>(d) *Cohort study* - If applicable, explain how loss to follow-up was addressed<br>*Case-control study* - If applicable, explain how matching of cases and controls was addressed<br>*Cross-sectional study* - If applicable, describe analytical methods taking account of sampling strategy<br>(e) Describe any sensitivity analyses			p. 9-11
Data access and cleaning methods		..		RECORD 12.1: Authors should describe the extent to which the investigators had access to the database population used to create the study population.	N/A

				RECORD 12.2: Authors should provide information on the data cleaning methods used in the study.	
Linkage		..		RECORD 12.3: State whether the study included person-level, institutional-level, or other data linkage across two or more databases. The methods of linkage and methods of linkage quality evaluation should be provided.	p. 8
**Results**					
Participants	13	(a) Report the numbers of individuals at each stage of the study (*e.g.*, numbers potentially eligible, examined for eligibility, confirmed eligible, included in the study, completing follow-up, and analysed)<br>(b) Give reasons for non-participation at each stage.<br>(c) Consider use of a flow diagram		RECORD 13.1: Describe in detail the selection of the persons included in the study (*i.e.*, study population selection) including filtering based on data quality, data availability and linkage. The selection of included persons can be described in the text and/or by means of the study flow diagram.	p. 12
Descriptive data	14	(a) Give characteristics of study participants (*e.g.*, demographic, clinical, social) and information on exposures and potential confounders<br>(b) Indicate the number of participants with missing data for each variable of interest<br>(c) *Cohort study* - summarise follow-up time			p. 12

		(*e.g.*, average and total amount)			
Outcome data	15	*Cohort study* - Report numbers of outcome events or summary measures over time<br>*Case-control study* - Report numbers in each exposure category, or summary measures of exposure<br>*Cross-sectional study* - Report numbers of outcome events or summary measures			p. 12-13
Main results	16	(a) Give unadjusted estimates and, if applicable, confounder-adjusted estimates and their precision (e.g., 95% confidence interval). Make clear which confounders were adjusted for and why they were included<br>(b) Report category boundaries when continuous variables were categorized<br>(c) If relevant, consider translating estimates of relative risk into absolute risk for a meaningful time period			p. 12-13
Other analyses	17	Report other analyses done—e.g., analyses of subgroups and interactions, and sensitivity analyses			N/A

**Discussion**					
Key results	18	Summarise key results with reference to study objectives			p. 15
Limitations	19	Discuss limitations of the study, taking into account sources of potential bias or imprecision. Discuss both direction and magnitude of any potential bias		RECORD 19.1: Discuss the implications of using data that were not created or collected to answer the specific research question(s). Include discussion of misclassification bias, unmeasured confounding, missing data, and changing eligibility over time, as they pertain to the study being reported.	p. 13
Interpretation	20	Give a cautious overall interpretation of results considering objectives, limitations, multiplicity of analyses, results from similar studies, and other relevant evidence			p. 13-16
Generalisability	21	Discuss the generalisability (external validity) of the study results			p. 14-16
**Other Information**					
Funding	22	Give the source of funding and the role of the funders for the present study and, if applicable, for the original study on which the present article is based			p. 5

Accessibility of protocol, raw data, and programming code		..		RECORD 22.1: Authors should provide information on how to access any supplemental information such as the study protocol, raw data, or programming code.	p. 12
